# Tilianin Ameliorates Cognitive Dysfunction and Neuronal Damage in Rats with Vascular Dementia via p-CaMKII/ERK/CREB and ox-CaMKII-Dependent MAPK/NF-*κ*B Pathways

**DOI:** 10.1155/2021/6673967

**Published:** 2021-09-04

**Authors:** Hailun Jiang, Ghulam Md Ashraf, Mimin Liu, Kaiyue Zhao, Yu Wang, Linlin Wang, Jianguo Xing, Badrah S. Alghamdi, Zhuorong Li, Rui Liu

**Affiliations:** ^1^Institute of Medicinal Biotechnology, Chinese Academy of Medical Sciences and Peking Union Medical College, Beijing 100050, China; ^2^Pre-Clinical Research Unit, King Fahd Medical Research Center, King Abdulaziz University, Jeddah, Saudi Arabia; ^3^Department of Medical Laboratory Technology, Faculty of Applied Medical Sciences, King Abdulaziz University, Jeddah, Saudi Arabia; ^4^Institute of Cardiovascular and Medical Sciences, BHF Glasgow Cardiovascular Research Center, University of Glasgow, 126 University Place, Glasgow G12 8TA, UK; ^5^Key Laboratory of Uighur Medicine of Xinjiang Uygur Autonomous Region, Xinjiang Institute of Materia Medica, Urumqi 830004, China

## Abstract

Vascular dementia (VaD) is a common cause of cognitive decline and dementia of vascular origin, but the precise pathological mechanisms are unknown, and so effective clinical treatments have not been established. Tilianin, the principal active compound of total flavonoid extract from *Dracocephalum moldavica* L., is a candidate therapy for cardio-cerebrovascular diseases in China. However, its potential in the treatment of VaD is unclear. The present study is aimed at investigating the protective effects of tilianin on VaD and exploring the underlying mechanism of the action. A model of VaD was established by permanent 2-vessel occlusion (2VO) in rats. Human neurons (hNCs) differentiated from human-induced pluripotent stem cells were used to establish an oxygen-glucose deprivation (OGD) model. The therapeutic effects and potential mechanisms of tilianin were identified using behavioral tests, histochemistry, and multiple molecular biology techniques such as Western blot analysis and gene silencing. The results demonstrated that tilianin modified spatial cognitive impairment, neurodegeneration, oxidation, and apoptosis in rats with VaD and protected hNCs against OGD by increasing cell viability and decreasing apoptosis rates. A study of the mechanism indicated that tilianin restored p-CaMKII/ERK1/2/CREB signaling in the hippocampus, maintaining hippocampus-independent memory. In addition, tilianin inhibited an ox-CaMKII/p38 MAPK/JNK/NF-*κ*B associated inflammatory response caused by cerebral oxidative stress imbalance in rats with VaD. Furthermore, specific CaMKII*α* siRNA action revealed that tilianin-exerted neuroprotection involved increase of neuronal viability, inhibition of apoptosis, and suppression of inflammation, which was dependent on CaMKII*α*. In conclusion, the results suggested the neuroprotective effect of tilianin in VaD and the potential mechanism associated with dysfunction in the regulation of p-CaMKII-mediated long-term memory and oxidation and inflammation involved with ox-CaMKII, which may lay the foundation for clinical trials of tilianin for the treatment of VaD in the future.

## 1. Introduction

Vascular dementia (VaD) is the second most common form of dementia, accounting globally for approximately 20% of dementia cases. It is caused by insufficient oxygen and nutrients due to a restricted blood supply to parts of the brain, leading to gradual cognitive decline. The prevalence of VaD in developing countries is approximately 5.3%, and the number of people suffering from VaD around the world is expected to double over the next 20 years [1–3]. However, no drugs have yet been developed that are effective at curing VaD.

It has been confirmed that a series of metabolic disorders caused by intracellular calcium overload are the leading causes of cognitive dysfunction in VaD [4]. Ca^2+^/calmodulin-dependent protein kinase II (CaMKII), a heteromeric serine/threonine-specific protein kinase, is a critical target enzyme in the Ca^2+^/CaM signaling system. Of the four CaMKII isoforms, *α*, *β*, *γ*, and *δ*, CaMKII*α* is the most abundant protein in the brain, playing a pivotal role in learning and memory [5]. There are two active forms of CaMKII, phosphorylated CaMKII (p-CaMKII) and oxidized CaMKII (ox-CaMKII). Intracellular calcium ions activate autophosphorylation of CaMKII, which plays a role in the induction and maintenance of long-term potentiation (LTP), referred to as a “molecular memory switch” [5]. Activated p-CaMKII upregulates downstream extracellular regulated protein kinase (ERK)/cAMP-response element-binding protein (CREB) pathway levels, triggering gene transcription and protein expression that enhance long-term memory formation and consolidation within the hippocampus [6]. Additionally, oxidative stress induced by intracellular calcium overload leads to the production of large quantities of reactive oxygen species (ROS), which then activates ox-CaMKII and triggers subsequent mitogen-activated protein kinase (MAPK)/nuclear factor kappa-B (NF-*κ*B) pathways, leading to inflammatory response, cell apoptosis, and ultimately neuronal damage [7]. Therefore, it can be inferred that CaMKII performs a pivotal role in many aspects of VaD involving the control of cognitive deficit and the evocation of oxidative stress, inflammatory reactions, and cell apoptosis.

Tilianin is the principal active ingredient extracted from the medicinal plant *Dracocephalum moldavica* L., an Uyghur medicine whose usage is supported by evidence-based medicine [8]. Previous research has demonstrated that tilianin displays cardiovascular protection; inhibits atherosclerosis, hypertension, diabetes, inflammation, and depression; and is an antioxidant, among its other properties [9–11]. As one of our ongoing studies, tilianin exerts beneficial effects on alleviating atherosclerosis lesions in vascular smooth muscle cells through inhibition of the TGF-*β*/Smad signaling, illustrating the potential protective effects of tilianin on vascular dysfunction [12, 13]. Furthermore, tilianin has been shown to protect the brain tissue of rats against acute cerebral ischemia-reperfusion [14]. Notably, the CaMKII-mediated apoptosis is found to play a role in the neuroprotection of tilianin against ischemic injury in a neuronal cell line [15]. Tilianin has thus become a preclinical candidate for the prevention of cardio-cerebrovascular disease supported by the “Significant New Drug Creation,” National Major Scientific and Technological Special Project of China. Therefore, elucidation of the therapeutic action of tilianin in VaD is of great importance.

In the present study, the efficacy of tilianin for the amelioration of cognitive dysfunction and neurodegenerative pathology in VaD was investigated *in vivo*. Moreover, the mechanisms underlying the neuroprotective action and antioxidant, anti-inflammatory, and antiapoptotic properties were elucidated.

## 2. Material and Methods

### 2.1. Materials

**Figure 1:**
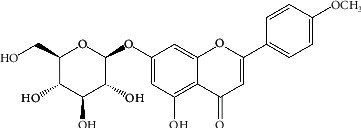
Chemical structure of tilianin.

Tilianin (Figure 1) was extracted from *Dracocephalum moldavica* L. A purity of 99% was obtained through a routine extraction process, as described previously [16].

### 2.2. Animals

Male, 7-week-old specific-pathogen-free Sprague Dawley (SD) rats (280 ± 20 g in weight) were obtained from Huafukang Biotechnology Co., Ltd. (Beijing, China). Five rats were housed in each cage with *ad libitum* access to food and water at a temperature of 22-24°C within a 12 h/12 h light/dark cycle. All experiments were approved by the Experimental Animal Care and Use Committee of the Institute of Medicinal Biotechnology (No. IMB-201807-D8-03).

### 2.3. Animal Model and Drug Treatment

**Figure 2:**
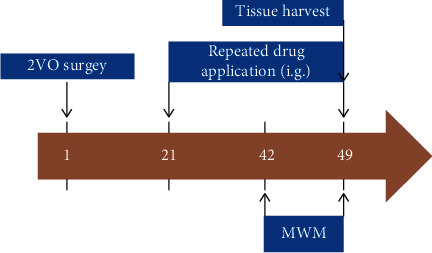
Scheme of the experimental procedures for establishment of the 2VO rat model and duration of tilianin administration. 2VO: 2-vessel occlusion; i.g.: intragastric administration; MWM: Morris water maze.

The 2-vessel occlusion (2VO) model is widely accepted to be an appropriate method of investigation of the molecular mechanisms of dementia induced by chronic cerebral ischemia and the evaluation of treatment efficacy [14, 17]. Briefly, following anesthetization using an intraperitoneal injection of pentobarbital sodium (30 mg/kg), the bilateral common carotid arteries were separated after creating an incision in the disinfected middle of the anterior neck in the rats after the fur was removed. The bilateral common carotid arteries were carefully double ligated with No. 0 surgical suture, avoiding damage to the cervical sympathetic and pneumogastric nerves. The same surgical procedure was performed on the sham group but without occlusion of the bilateral common carotid arteries. Penicillin was administered by intramuscular injection after surgery to prevent infection. A total of fifty rats underwent surgery and after 21 days were randomly allocated into four groups, as directed by a random number generator in Microsoft Excel, including a sham group (*n* = 14), 2VO model group (*n* = 12), and 2VO groups treated with 20 mg/kg tilianin (*n* = 12) and 40 mg/kg (*n* = 12). The rats in the treatment groups received a daily dose of tilianin dissolved in 0.5% carboxymethylcellulose sodium (CMC-Na) by oral administration for four weeks, as described previously in the literature [14]. Rats in the sham and model groups received the 0.5% CMC-Na vehicle in the same way. No adverse conditions were experienced when conducting the animal studies. The *in vivo* experimental protocol is displayed in Figure 2.

### 2.4. Morris Water Maze Test

The Morris water maze (MWM) test is the most commonly used procedure for the evaluation of brain function in relation to spatial cognition [18]. The MWM test was performed each day over the last six days of tilianin treatment, including the location of navigation for five days and a probe trial on the sixth day. For the navigation test, the rats were tested on four occasions each day from one starting point in each of four quadrants. In each trial, the rats searched for an underwater platform for a maximum of 60 seconds. The time taken to reach the platform was recorded as the escape latency. Rats that did not find the platform within 60 seconds were guided to the platform, where they were allowed to remain for an additional 10 seconds. The probe trial was performed on the sixth day to assess memory about platform location. Rats were released from the opposite quadrant into the tank without the platform and were observed swimming freely for 60 seconds. The duration within the target quadrant and crossing through that platform location was recorded.

### 2.5. Histological Examination

Fluoro-Jade B (FJB) and Nissl staining were used to assess neuronal degeneration. FJB staining is commonly used for labeling degenerated neurons in *ex vivo* central nervous system tissue [19]. Nissl staining exhibits selective affinity for Nissl bodies, the large granular structures containing rough endoplasmic reticulum (RER) and extranuclear RNA that are found in neurons, which disappear when experiencing a variety of pathological conditions [20]. Briefly, rats were sacrificed after behavioral testing by intraperitoneal injection of pentobarbital sodium (60 mg/kg), then perfused with phosphate-buffered saline (PBS) and 4% paraformaldehyde (PFA). Sections (4 *μ*m) of brain tissue were mounted on slides, rinsed with PBS for 2 minutes, incubated in 0.06% potassium permanganate solution for 5 minutes, rinsed with water for 1 minute, and finally stained with 0.0004% FJB solution (Histochem, Jefferson, AR, USA) for 20 minutes protected from light at room temperature (RT). For Nissl staining, slides were incubated in Nissl stain (Servicebio, Wuhan, China) at 50°C for 20 minutes, rinsed with distilled water then dehydrated in 95% ethanol. Finally, the slides were cleared in xylene and mounted using dibutyl polystyrene xylene. Images were acquired using a fluorescence microscope (Olympus, Tokyo, Japan).

### 2.6. Western Blot Analysis

Western blot analysis was conducted using routine protocols. Briefly, hippocampal and cortical tissues were independently homogenized with RIPA buffer (New Cell and Molecular Biotech Co., Ltd., Suzhou, China) containing protease and phosphatase inhibitors (Topscience, Shanghai, China), after which they were centrifuged at freezing temperatures at 13000 g for 15 minutes. Total protein concentration was measured using a BCA assay (CWBio, Beijing, China). Twenty *μ*g protein was analyzed in each assay, as described in the published literature [16]. The primary antibodies used in the analysis are displayed in Table 1. Protein bands were acquired on a Fusion-FX6 imaging system (Vilber Lourmat, Marne-la-Valle, France). GAPDH was used as a loading control.

### 2.7. Human iPSC Culture, NSC Expansion, and NC Differentiation

Human-induced pluripotent stem cells (hiPSCs) (originating from human renal epithelial cells, Cellapy Biotechnology, Beijing, China) were maintained in PSCeasy®II hiPSC complement medium (Cellapy Biotechnology) in a humidified incubator at 37°C and 5% CO_2_. When 80% confluent, the hiPSCs were passaged using digestion solution (Cellapy Biotechnology). The hiPSCs were induced to differentiate into human neural stem cells (hNSCs) [21, 22] when the hiPSCs had become 100% confluent. Three specific markers of hNSCs (Nestin, paired box protein 6 (PAX6), and SRY-box transcription factor 2 (Sox2)) were used to identify hNSCs in the present study (Figure S1(a)). hNSCs were passaged more than three times for subsequent experiments. After digestion with Cellapy Biotechnology digestion solution, hNSCs were cultured at a density of 5 × 10^3^ cells/mL in NeuroEasy human nerve cell differentiation medium for 21 days. Three specific markers of human neurons (hNCs) differentiated from hNSCs, microtubule-associated protein-2 (MAP2), *β*-tubulin III, and neuron-specific enolase (NSE) were used to identify cells with a neuronal phenotype (Figure S1(b)).

### 2.8. Tilianin Treatment and siRNA Transfection

**Figure 3:**
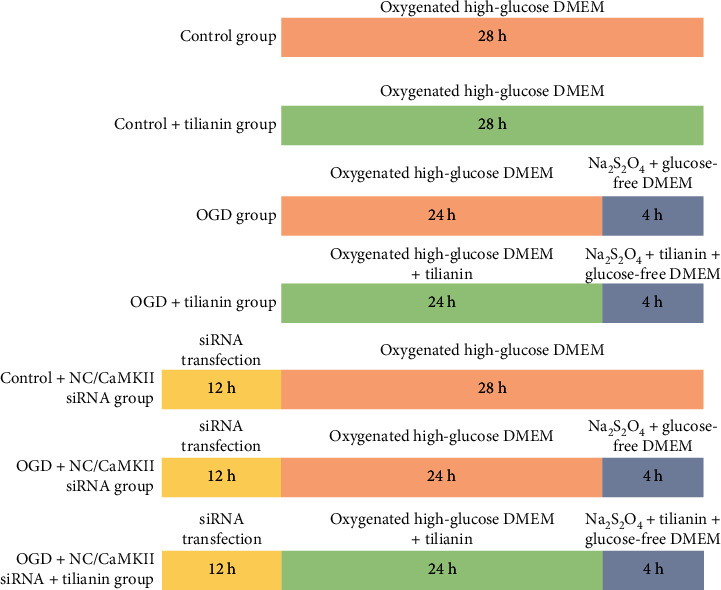
Scheme of the experimental procedures for the OGD model establishment, CaMKII*α* siRNA transfection, and tilianin administration in hNCs. NC: negative control; OGD: oxygen-glucose deprivation.

In accordance with our previous study [15], sodium dithionite (Na_2_S_2_O_4_) was used to establish an *in vitro* oxygen-glucose deprivation (OGD) model to simulate pathological cerebral ischemia. OGD was imposed on hNCs using 800 *μ*M Na_2_S_2_O_4_ for 4 h after which the hNCs were randomly allocated into two groups: a high-glucose control group and a Na_2_S_2_O_4_-injury group, encompassing 0 *μ*M, 3 *μ*M, 10 *μ*M, and 30 *μ*M tilianin treatment groups. The *in vitro* experimental protocol is displayed in Figure 3.

To study the mechanism of action, CaMKII*α* siRNA was synthesized by Sangon Biotech (Shanghai, China), as presented in Table 2. The hNCs were allocated into negative control (NC) transfection and CaMKII*α* siRNA transfection groups, which were further divided into 0 *μ*M, 3 *μ*M, 10 *μ*M, and 30 *μ*M tilianin treatment subgroups. siRNAs were transfected into cells using INTERFERin (Polyplus-transfection, New York, NY, USA) for 12 h, after which the hNCs were treated with tilianin for 24 h; then, OGD injury was performed. In addition, siRNA transfection was verified by quantitative polymerase chain reaction (qPCR) and Western blot analysis in accordance with previously published literature [16], as shown in Table S1 and Figure S2.

### 2.9. Cell Viability Assay

After the treatment described above, hNC viability was determined using a CellCounting-Lite™ 2.0 Luminescent cell viability assay (CCL, Vazyme Biotech, Nanjing, China) in accordance with the manufacturer's instructions. Luminescence was measured using a SPARK 20 M microplate reader (Tecan Group Ltd., Männedorf, Switzerland).

### 2.10. Detection of Apoptosis

A TdT-mediated dUTP Nick End Labeling (TUNEL) BrightRed Apoptosis detection kit (Vazyme Biotech) was used to determine the degree of apoptosis in hNCs in accordance with the manufacturer's instructions. Image acquisition and analysis were conducted using a microplate reader Spark Cyto (Tecan Group Ltd.).

### 2.11. Superoxide Dismutase (SOD), Glutathione Peroxidase (GSH-Px), and Malondialdehyde (MDA) Measurement

SOD, GSH-Px, and MDA activities in homogenates of the cortex and hNCs were separately measured using commercial assay kits (Nanjing Jiancheng Bioengineering Institute, Nanjing, China; KeyGen BioTECH, Nanjing, China) in accordance with the manufacturer's guidelines [23, 24].

### 2.12. Cellular Immunofluorescence

Cellular immunofluorescence assays were performed to detect the transduction of CaMKII-associated signaling pathways, as described previously [15]. After incubation with appropriate primary antibodies (Table 3) overnight, hNCs were incubated with a secondary antibody (goat anti-rabbit conjugated with Alexa Fluor 488, 1 : 200, Servicebio; or goat anti-mouse conjugated with Alexa Fluor 488, 1 : 1000, Thermo Fisher Scientific, Carlsbad, CA, USA) at RT for 2 h. Images and fluorescence levels were acquired and analyzed using a Spark Cyto microplate reader (Tecan Group Ltd.) and Cellomics ArrayScan^VTI^ HCS Reader (Thermo Fisher Scientific Cellomics, Waltham, MA, USA), respectively.

### 2.13. Caspase-3 Activity Assay

Caspase-3 activity in homogenates of the hippocampus and hNCs was quantified using a caspase-3 activity assay kit (Abcam, Cambridge, MA, USA).

### 2.14. Enzyme-Linked Immunosorbent Assay (ELISA)

The levels of tumor necrosis factor-alpha (TNF-*α*), interleukin-6 (IL-6), 3-nitrotyrosine (3-NT), 4-hydroxynonenal (4-HNE), and interleukin-1*β* (IL-1*β*) in the homogenates of the cortex and in the supernatant of hNCs were measured using corresponding ELISA kits (Proteintech, Rosemont, IL, USA; RayBiotech, Inc., Norcross, GA, USA; Nanjing Jiancheng Bioengineering Institute) [25–29]. Data are presented as pg/mL.

### 2.15. Statistical Analysis

Data are expressed as means ± standard deviation (SD). Escape latency and speed of swimming in the MWM test were analyzed using IBM SPSS Statistics version 25.0 software (IBM, Armonk, NY, USA) by analysis of variance (ANOVA) for repeated measures, with Tukey's *post hoc* analyses between groups. Other statistical analyses were conducted using the GraphPad Prism version 8.0 software (GraphPad Inc., La Jolla, CA, USA) by one-way ANOVA and student's *t*-tests. *P* < 0.05 was considered statistically significant.

## 3. Results

### 3.1. Treatment with Tilianin Improved Spatial Learning and Memory Capability in Rats with VaD

**Figure 4:**
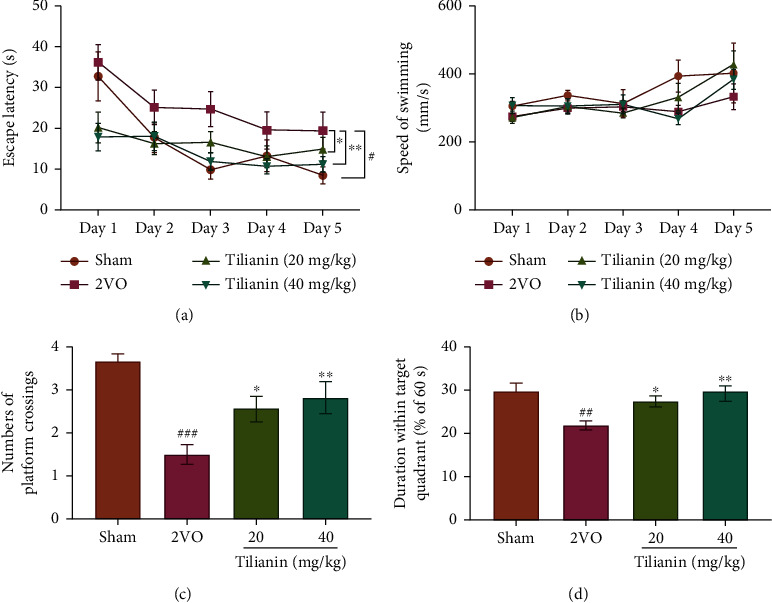
Tilianin treatment improved spatial cognition in rats with VaD: (a) escape latency in the MWM navigation test; (b) swimming speed in the navigation test; (c) increase in the number of platform crossings for tilianin-treated rats with 2VO in the probe test; (d) increase in the duration within the target quadrant of tilianin-treated rats with 2VO in the probe test. Results represent means ± SD, *n* = 12~14. ^#^*P* < 0.05, ^##^*P* < 0.01, ^###^*P*<0.001 vs. sham group; ^∗^*P* < 0.05, ^∗∗^*P* < 0.01 vs. 2VO group.

In the navigation test, escape latency in rats is an indicator of spatial learning capability. The performance of the rats in every group improved, with shortened escape latency during the five-day training process (Figure 4(a), *F*_(4,184)_ = 14.301, *P* < 0.001). Different treatments resulted in varying spatial learning capability (Figure 4(a), *F*_(3, 46)_ = 3.739, *P* < 0.05). Subsequent comparisons demonstrated that rats with 2VO displayed longer escape latency compared with sham rats (Figure 4(a), *P* < 0.05), and dosages of both 20 mg/kg and 40 mg/kg tilianin significantly reduced escape latency on days 2 to 5 compared with rats with 2VO (Figure 4(a), *P* < 0.05 and 0.01). However, the speed of swimming over the five days was not significantly different between groups (Figure 4(b)), indicating that there was no impairment of motor function in any group.

In the probe trial, the duration that rats were in the target quadrant and passing across the original location of the platform were two indicators of spatial memory capability. The rats with 2VO made fewer crossings over the location of the escape platform and less time swimming within the target quadrant than the sham rats (Figures 4(c) and 4(d), *P* < 0.001 and 0.01, respectively). Nevertheless, the rats with 2VO treated with 20 mg/kg and 40 mg/kg tilianin displayed significantly increased numbers of crossings, and the duration within the target quadrant was prolonged compared with untreated rats (Figures 4(c) and 4(d), *P* < 0.05-0.01). These results indicate that rats with 2VO displayed significant spatial learning and memory impairments, while treatment with tilianin was effective at rescuing these cognitive deficits.

### 3.2. Tilianin Treatment Alleviated Neurodegeneration in Rats with VaD

**Figure 5:**
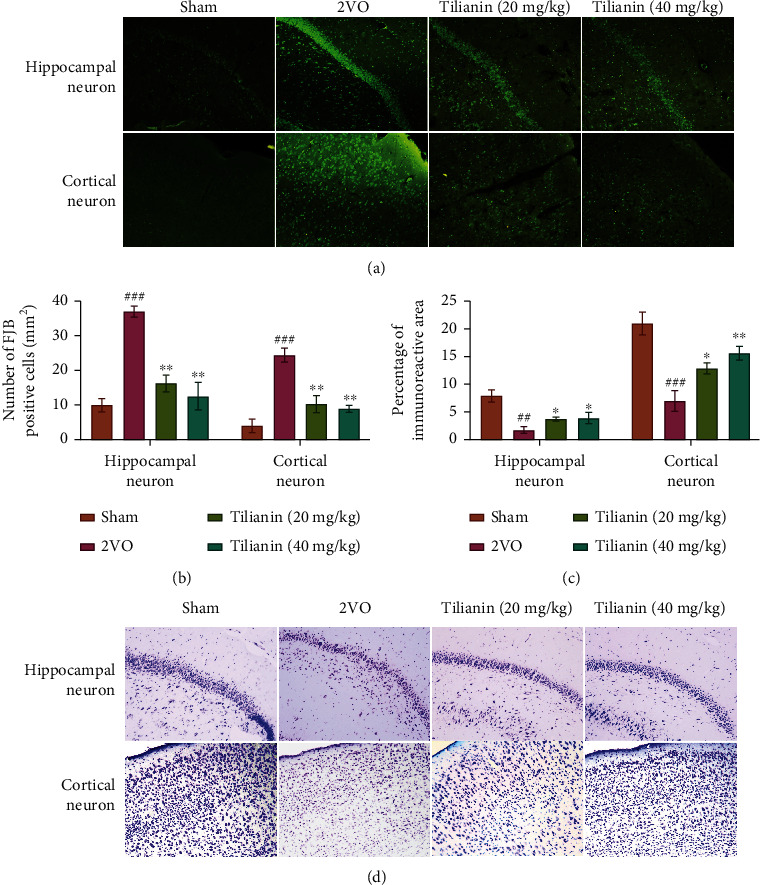
Tilianin treatment alleviated neurodegeneration in rats with VaD: (a) representative images of FJB stained hippocampal and cortical neurons (200x); (b) mean number of FJB-positive cells/mm^2^; (c) mean number of Nissl bodies of Nissl-stained hippocampus and cortex; (d) representative images of Nissl-stained hippocampal and cortical neurons (200x). Results represent means ± SD. *n* = 3. ^##^*P* < 0.01, ^###^*P* < 0.001 vs. sham group; ^∗^*P* < 0.05, ^∗∗^*P* < 0.01 vs. 2VO group.

In the present study, two parameters of neuronal degeneration in injured rats were measured to determine the cerebral protective effects of tilianin on rats with 2VO. The cell bodies, dendrites, axons, and axon terminals of degenerating neurons have a strong affinity with FJB dye, whereas healthy neurons do not. As shown in Figures 5(a) and 5(b), the hippocampal and cortical neurons in rats with 2VO displayed a bright green fluorescence intensity representing extensive FJB labeling compared with sham rats (*P* < 0.001). However, the number of FJB-positive neurons was significantly decreased in rats with 2VO treated with tilianin in a dose-dependent manner (all *P* < 0.01), demonstrating that tilianin treatment ameliorated neuronal degeneration.

Similar changes were observed in Nissl-stained sections. In the sham group, neurons exhibited normal morphology with transparent Nissl bodies in the cytoplasm (Figure 5(d)). In contrast, a significant reduction in the number of Nissl bodies was observed in tissue from the hippocampus and cortex of rats in the 2VO group (Figures 5(c) and 5(d), *P* < 0.01 and 0.001). Compared with rats with 2VO, the number of Nissl bodies in tilianin-treated rats suffering from 2VO increased in a dose-dependent manner (Figures 5(c) and 5(d), *P* < 0.05-0.01). Considering that Nissl bodies are involved in the synthesis of neuronal proteins [18], the results demonstrate that tilianin was able to reverse the neuronal damage caused by chronic vascular injury in the 2VO model.

### 3.3. Tilianin Restored Long-Term Memory-Related p-CaMKII/ERK/CREB Pathway Signaling and Inhibited the Apoptotic Pathway in the Hippocampus of Rats with VaD

**Figure 6:**
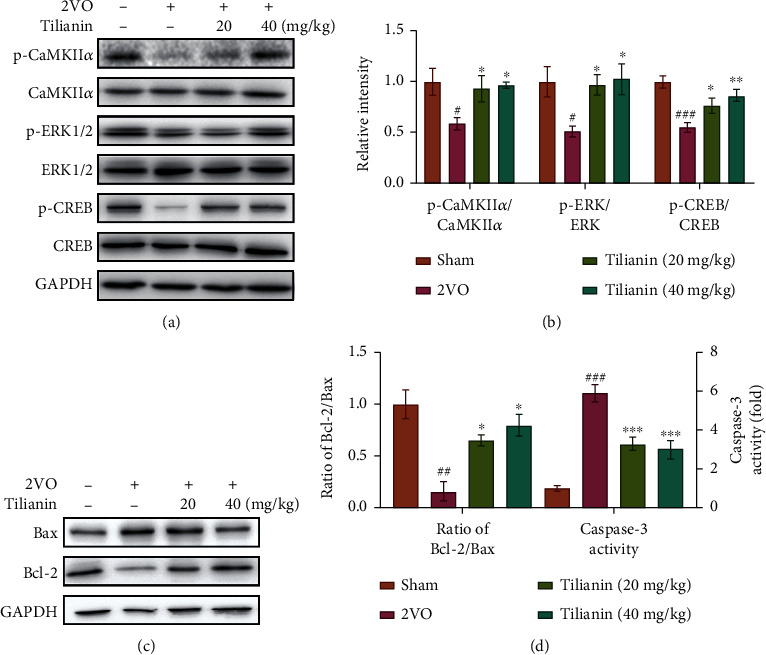
Tilianin restored long-term memory-related protein expression through p-CaMKII/ERK/CREB pathway in the hippocampus of rats with VaD. (a) Representative Western blot bands for p-CaMKII, total CaMKII, p-ERK1/2, total ERK1/2, p-CREB, and total CREB in the different groups. (b) Quantitative analysis of ratios of p-CaMKII/CaMKII, p-ERK/ERK, and p-CREB/CREB as observed in Western blots. (c) Representative Western blot bands of Bax and Bcl-2 in the different groups. (d) Quantitative analysis of the ratio of Bcl-2/Bax observed in Western blots and caspase-3 activity in the hippocampus. Results represent means ± SD, *n* = 5. ^##^*P* < 0.01, ^###^*P* < 0.001 vs. sham group; ^∗^*P* < 0.05, ^∗∗^*P* < 0.01, ^∗∗∗^*P* < 0.001 vs. 2VO group.

To further elucidate the potential mechanism by which tilianin restored long-term memory, the expression of proteins with which it was associated was measured by Western blot analysis. As shown in Figures 6(a) and 6(b), the ratio of p-CaMKII/CaMKII, p-ERK/ERK, and p-CREB/CREB in the hippocampus of rats with 2VO was lower than in sham rats (*P* < 0.05-0.001). Treatment with tilianin resulted in an increase in the ratio of p-CaMKII/CaMKII (*P* < 0.05), accompanied by upregulation of the ratio of p-ERK/ERK and p-CREB/CREB in the hippocampus (*P* < 0.05-0.01). These results suggest that the CaMKII/ERK/CREB signaling might contribute to tilianin-mediated improvement in cognition.

As expected, tilianin treatment inhibited apoptosis by elevation of the B cell lymphoma-2 (Bcl-2)/Bcl-2 associated X (Bax) protein ratio and reduction of the caspase-3 activity in the hippocampus of the rats with 2VO (Figures 6(c) and 6(d), *P* < 0.05-0.001), consistent with previous observations that tilianin treatment alleviated neuronal degeneration, as demonstrated by FJB and Nissl staining.

### 3.4. Tilianin Prevented Oxidative Stress and ox-CaMKII-Mediated Inflammation in the Prefrontal Cortex of Rats with VaD

**Figure 7:**
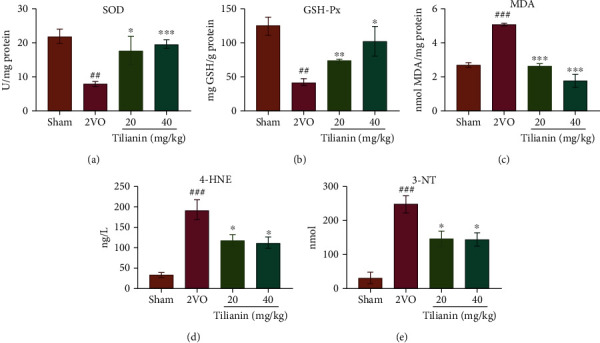
Tilianin inhibited the oxidative stress in the prefrontal cortex of rats with VaD. (a–e) SOD (a), GSH-Px (b), MDA (c), 4-HNE (d), and 3-NT(e) activity in cortex homogenates of different groups (*n* = 4). Results represent means ± SD. ^##^*P* < 0.01, ^###^*P* < 0.001 vs. sham group; ^∗^*P* < 0.05, ^∗∗^*P* < 0.01, ^∗∗∗^*P* < 0.001 vs. 2VO group.

Ox-CaMKII can be formed by oxidative stress, and such levels are elevated in VaD. Levels of the antioxidant enzymes SOD and GSH-Px in the supernatant of homogenates of the cortex of rats with 2VO were significantly lower than those of the sham group (Figures 7(a) and 7(b), both *P* < 0.01), while lipid peroxidation production MDA and 4-HNE were significantly higher than those of the sham group (Figures 7(c) and 7(d), both *P* < 0.001), and the protein oxidative damage product 3-NT was significantly increased compared with the sham group (Figure 7(e), *P* < 0.001). However, tilianin treatment resulted in significantly increased levels of SOD and GSH-Px and decreased levels of MDA, 4-HNE, and 3-NT (*P* < 0.05-0.001). This indicates that tilianin exerted antioxidant effects by upregulation of the antioxidant enzyme system and downregulation of oxidative products.

**Figure 8:**
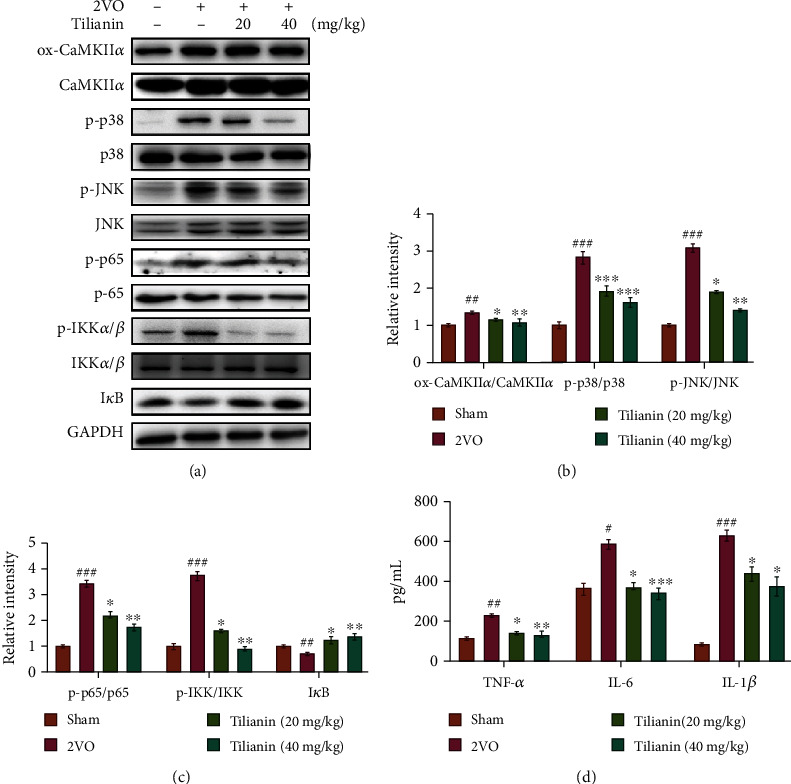
Tilianin prevented neuronal inflammation in the prefrontal cortex of rats with VaD. (a) Representative Western blot bands of ox-CaMKII, total CaMKII, p-p38, total p38, p-JNK, total JNK, p-p65, total p65, p-IKK*α*/*β*, total IKK*α*/*β*, and I*κ*B in the different groups. (b) Quantitative analysis of the ratios of ox-CaMKII/CaMKII, p-p38/p38, p-JNK/JNK, and p-p65/p65 observed in Western blots (*n* = 5). (c) Quantitative analysis of the ratios of p-p65/p65, p-IKK/IKK, and I*κ*B observed in Western blots (*n* = 5). (d) Secretion of TNF-*α*, IL-6, and IL-1*β* as detected by ELISA (*n* = 4). Results represent means ± SD. ^#^*P* < 0.05, ^##^*P* < 0.01, ^###^*P* < 0.001 vs. sham group; ^∗^*P* < 0.05, ^∗∗^*P* < 0.01, ^∗∗∗^*P* < 0.001 vs. 2VO group.

Subsequent results demonstrated that the ratio of ox-CaMKII/CaMKII was significantly greater in rats with 2VO (Figures 8(a) and 8(b), *P* < 0.01), with the high levels of ox-CaMKII activating the expression of downstream ratio of p-p38/p38 and p-c-Jun N-terminal kinase (JNK)/JNK (Figures 8(a) and 8(b), both *P* < 0.001), triggering the activation of NF-*κ*B p65 (p-p65/p65) and inhibitor of I*κ*B kinase (p-IKK*α*/*β*/IKK*α*/*β*) and the degeneration of inhibitor of NF-*κ*B (I*κ*B) (Figures 8(a) and 8(c), *P* < 0.01-0.001) that further promoted a massive release of the inflammatory factors TNF-*α*, IL-6, and IL-1*β* (Figure 8(d), *P* < 0.05-0.001). However, in tilianin-treated rats with 2VO, the ox-CaMKII/p38 MAPK/JNK/p65 activated inflammatory signaling pathways were effectively inhibited (Figures 8(a)–8(d), *P* < 0.05-0.001). These results indicate that tilianin treatment suppressed the oxidative stress, and on the other hand, inhibited the expression of ox-CaMKII, thereby controlling multiple activations of the downstream MAPK/NF-*κ*B signaling pathways and exerting neuroprotective effects.

### 3.5. Tilianin Exerted Neuroprotection on OGD-Injured hNCs

**Figure 9:**
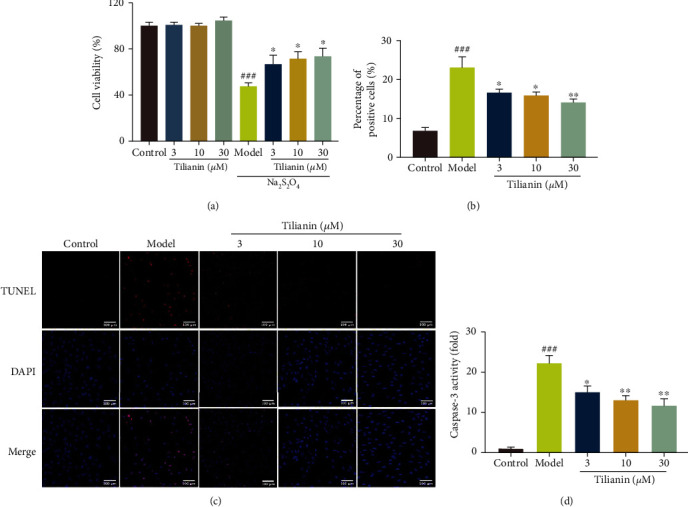
Tilianin provided neuroprotection to OGD-injured hNCs. (a) Increased viability of OGD-injured hNCs treated with tilianin as evaluated by CCL assay (*n* = 6). (b) Decreased percentage of TUNEL-positive cells in OGD-injured hNCs treated by tilianin. (c) Representative images of TUNEL staining. Bar: 100 *μ*m. (d) Decreased caspase-3 activity in OGD-injured hNCs treated with tilianin. Results represent means ± SD. ^###^*P* < 0.001 vs. control group; ^∗^*P* < 0.05, ^∗∗^*P* < 0.01 vs. OGD group.

To verify the pharmacological action of tilianin on humans, its neuroprotective effects on hNCs from OGD injury were further explored. Firstly, as shown in Figure 9(a), OGD injured hNCs by decreasing cell viability (*P* < 0.001), while tilianin rescued cell viability in a concentration-dependent fashion (3 *μ*M, 10 *μ*M, and 30 *μ*M) against OGD injury (Figure 9(a), *P* < 0.05). In addition, tilianin did not influence the survival of normally cultured hNCs at these tested concentrations (Figure 9(a), *P* = 0.94, 0.99, and 0.33).

The degree of hNC apoptosis was evaluated using TUNEL staining, the results of which are displayed in Figures 9(b) and 9(c). The proportion of TUNEL-positive cells in OGD-injured hNCs was significantly greater than the control group (*P* < 0.001), while treatment with tilianin reduced the proportion of TUNEL-positive cells in a concentration-dependent manner (Figures 9(b) and 9(c), *P* < 0.05-0.01). Additionally, increased caspase-3 activity in response to OGD injury was alleviated by tilianin at these administered concentrations (Figure 9(d), *P* < 0.05-0.01). Thus, tilianin protected against OGD-injury via reduced neuronal apoptosis.

### 3.6. Tilianin Inhibited Oxidative Stress on OGD-Injured hNCs

**Figure 10:**
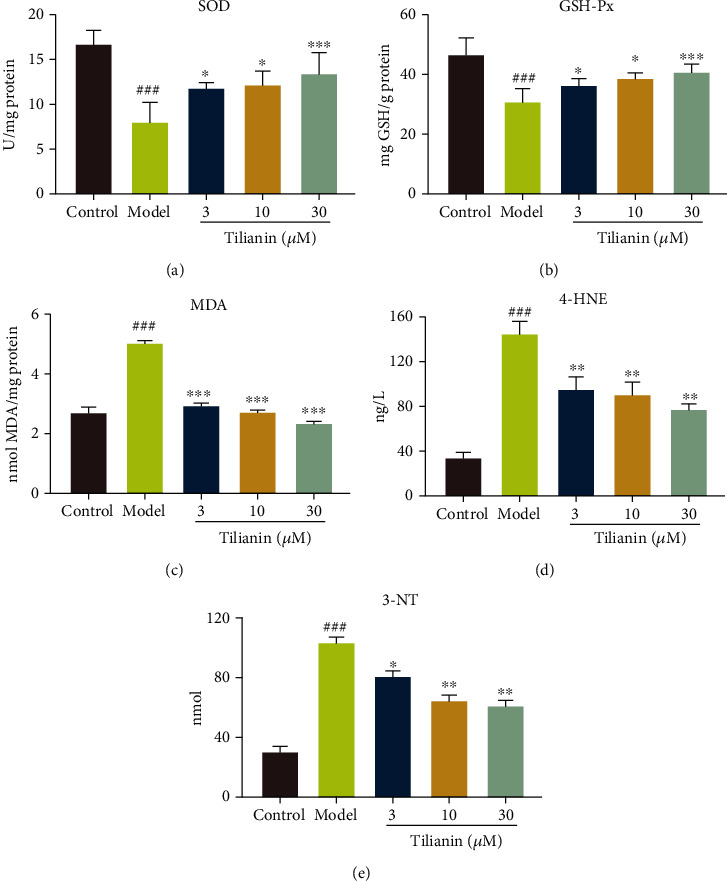
Tilianin inhibited oxidative stress on OGD-injured hNCs. (a–e) Activity of SOD (a), GSH-Px (b), MDA (c), 4-HNE (d), and 3-NT (e) in OGD-injured hNCs in the different groups (*n* = 4). Results represent means ± SD. ^###^*P* < 0.001 vs. control group; ^∗^*P* < 0.05, ^∗∗^*P* < 0.01, ^∗∗∗^*P* < 0.001 vs. OGD group.

Corresponding to the results *in vivo*, we detected the effect of tilianin on oxidative stress markers in OGD-injured hNCs. The results demonstrated that tilianin increased the activity of antioxidant enzymes SOD and GSH (Figures 10(a) and 10(b), *P* < 0.05-0.001) while decreasing the levels of MDA, 4-HNE, and 3-NT (Figures 10(c)–10(e), *P* < 0.05-0.001), exerting the similar antioxidative effect *in vivo* and *in vitro*.

### 3.7. Tilianin Protected OGD-Injured hNCs via Inhibition of CaMKII*α*

**Figure 11:**
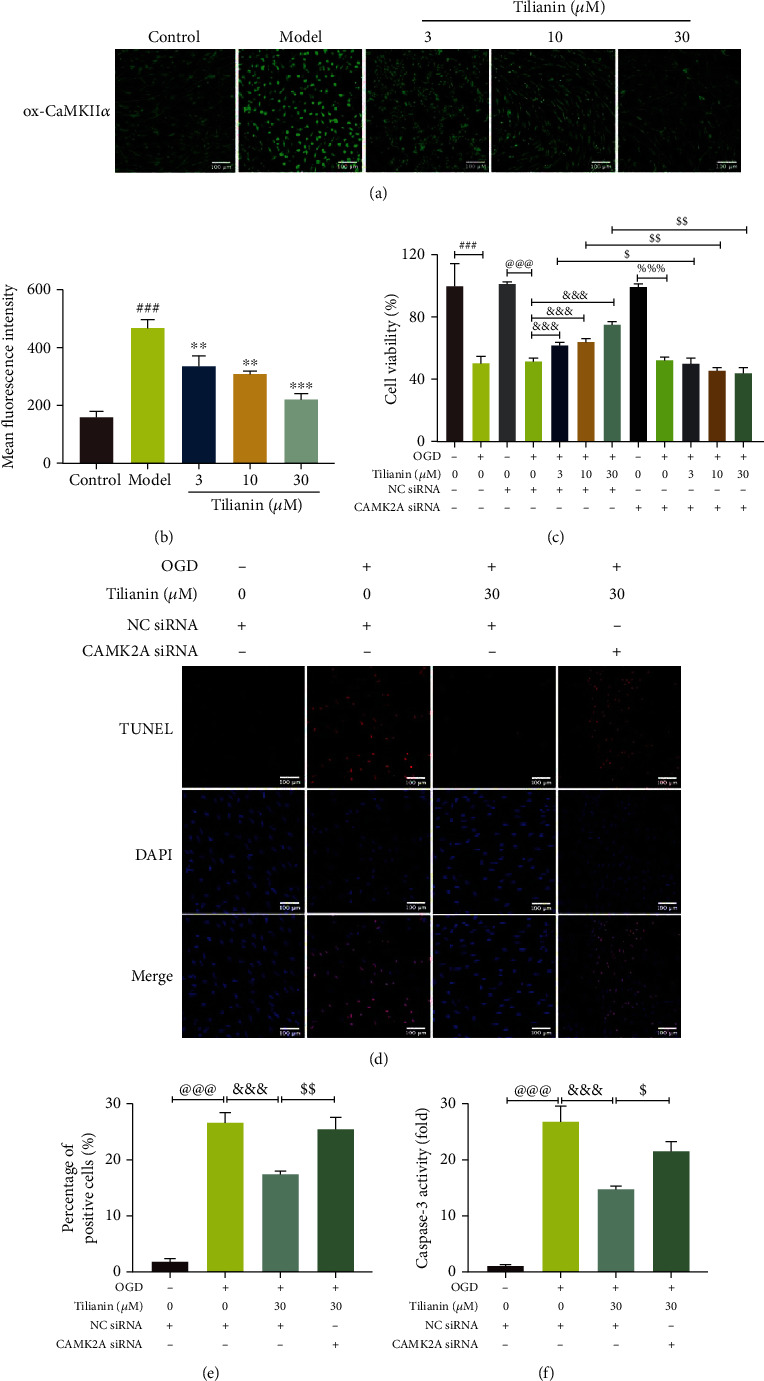
Tilianin protection of hNCs against OGD depended on inhibition of CaMKII*α*. (a) Representative immunofluorescence images of ox-CaMKII positivity. Bar: 100 *μ*m. (b) Mean fluorescence intensity of ox-CaMKII (*n* = 4). (c) Decreased viability of OGD-injured hNCs treated with tilianin in the presence of CaMKII*α* siRNA. (d) Representative images of TUNEL staining. Bar: 100 *μ*m. (e) Increased apoptosis of OGD-injured hNCs treated with tilianin in the presence of CaMKII*α* siRNA. (f) Increased caspase-3 activity of OGD-injured hNCs treated with tilianin in the presence of CaMKII*α* siRNA. Results represent means ± SD. ^###^*P* < 0.001 vs. control; ^∗∗^*P* < 0.01, ^∗∗∗^*P* < 0.001 vs. OGD; ^@@@^*P* < 0.001 vs. control + NC siRNA; ^&&&^*P* < 0.001 vs. OGD + NC siRNA; ^%%%^*P* < 0.001 vs. control + CAMK2A siRNA; ^$^*P* < 0.05, ^$$^*P* < 0.01 vs. OGD + NC siRNA + tilianin.

Based on previous research, tilianin is known to weakly interact with CaMKII*α* either in binding score or inhibitory activity and functions as a moderate inhibitor of the CaMKII*α* expression [15]. Consistent with previous observations, tilianin strongly inhibited the upregulated ox-CaMKII expression induced by OGD in hNCs with increasing concentration (Figures 11(a) and 11(b), *P* < 0.01-0.001).

Furthermore, to determine whether inhibition of the CaMKII*α* expression contributed to tilianin-mediated neuroprotection against OGD-induced cytotoxicity, CaMKII*α* siRNA was used to silence the expression of CaMKII*α*. As shown in Figures 11(c)–11(f), due to CaMKII*α* siRNA, both the ability of tilianin to rescue cell viability and inhibit apoptosis were blocked after exposure to OGD at every administered concentration (*P* < 0.05-0.01). Besides, NC siRNA did not affect the cell viability of hNCs either in the control group or in the OGD group (Figure 11(c), *P* = 0.88 and 0.81).

**Figure 12:**
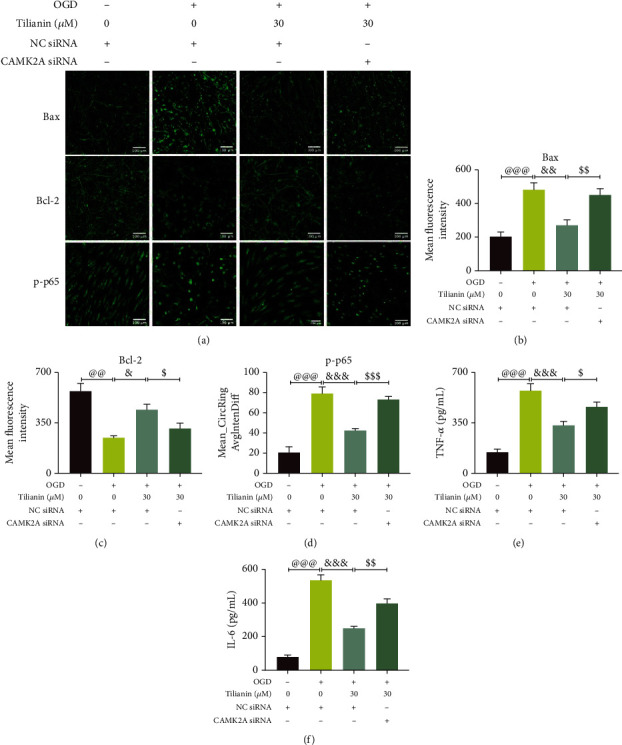
Tilianin inhibited CaMKII*α*-dependent intrinsic apoptosis and the inflammatory signaling pathway of OGD-injured hNCs but was blocked by the action of CaMKII*α* siRNA. (a) Representative immunofluorescence images of Bax, Bcl-2, and p-p65. Bar: 100 *μ*m. (b, c) Mean fluorescence intensity of Bax (b) and Bcl-2 (c) (*n* = 4). (d) Increased activity of p-p65 of OGD-injured hNCs treated with tilianin after the addition of CaMKII*α* siRNA. (e, f) Increased expression of TNF-*α* (e) and IL-6 (f) in OGD-injured hNCs treated with tilianin after the addition of CaMKII*α* siRNA. Results represent means ± SD. ^@@^*P* < 0.01, ^@@@^*P* < 0.001 vs. control + NC siRNA; ^&^*P* < 0.05, ^&&^*P* < 0.01, ^&&&^*P* < 0.001 vs. OGD + NC siRNA; ^$^*P* < 0.05, ^$$^*P* < 0.01, ^$$$^*P* < 0.001 vs. OGD + NC siRNA + tilianin.

In addition to neuronal cell viability, the intrinsic apoptotic and inflammatory signaling pathways were measured based on the *in vivo* results. Similarly, CaMKII*α* silencing suppressed tilianin-mediated inhibition of neuronal apoptosis via elimination of the restoration of Bax/Bcl-2 balance (Figures 12(a)–12(c), *P* < 0.05-0.01). The inhibitory effect of tilianin on the inflammatory pathway was correspondingly weakened in response to the action of CaMKII*α* siRNA, represented by the rebounding of p-p65 translocation and the increased levels of TNF-*α* and IL-6 (Figures 12(a), 12(d)–12(f), *P* < 0.05-0.001). Collectively, these results suggest that the neuroprotective effects of tilianin against OGD injury may depend on CaMKII*α* inhibition involving intrinsic apoptosis and inflammatory pathways.

## 4. Discussion

The present study demonstrated the neuroprotective effects and underlying mechanisms of tilianin on rats with VaD and OGD-injured hNCs. The results suggest that tilianin treatment ameliorated spatial learning and memory impairment, neurodegeneration, and the occurrence of pathological oxidative stress in rats with 2VO, in addition to neuronal apoptosis and inflammation both in rats with 2VO and OGD-injured hNCs. Further investigation *in vivo* revealed that tilianin treatment maintained long-term memory-related signaling molecules by increasing p-CaMKII/ERK/CREB signaling transduction in the hippocampus of rats with 2VO. Additionally, tilianin treatment regulated the inflammatory response through the p38 MAPK/JNK/NF-*κ*B signaling pathway by inhibition of oxidative stress and downregulation of the ox-CaMKII expression. These observations indicated that tilianin might exert neuroprotection against VaD by acting on two active forms of CaMKII.

Long-term cerebral ischemia causes neuronal apoptosis, neurodegeneration, and structural damage in brain tissue, resulting in a decline in cognitive capability, eventually developing into VaD [30]. In the present study, 2VO injury caused apparent pathological abnormalities, characterized by the significantly increased number of degenerative neurons in the brain, as indicated by FJB staining. In addition, the number of Nissl bodies in the hippocampus and cortex was substantially reduced, indicating that neurons were damaged by chronic ischemia in the rats with 2VO, finally manifesting as learning and memory dysfunction.

Tilianin is a principal active ingredient of the total flavonoid extract from *Dracocephalum moldavica* L., a traditional Uyghur medicine that has been demonstrated to improve spatial learning and memory capability in a transgenic AD mouse model [31]. Some of the vascular lesions, such as atherosclerosis of blood vessels, are considered to be risk factors in the process of cognitive impairment, among which endothelial dysfunction causes aberrant proliferation and migration of the adjacent VSMCs, resulting in vascular function and structure changes, eventually leading to cognitive impairment in VaD [32]. Our previous studies have illustrated that tilianin inhibits the proliferation and migration of rat VSMCs via suppressing the TGF-*β*/Smad pathway, thereby improving vascular function against atherosclerosis [12, 13]. These previous findings have provided evidence for the cardiovascular protective effects of tilianin that contribute to the potential benefit against VaD. Although tilianin has displayed neuroprotection against ischemic injury in our previous studies, in an OGD cell line model [15] and a rat model of cerebral ischemia-reperfusion injury [14], it is unclear the neuroprotective role and mechanisms of tilianin in alleviating cognitive deficits induced by VaD.

In the present study, rats with 2VO had impairment in spatial memory demonstrated by longer escape latency time and lower number of platform crossings than the sham group. Rats with 2VO receiving 20 mg/kg and 40 mg/kg tilianin treatment exhibited improved cognitive performance in the MWM test, represented by reduced swimming latency, increased crossings, and extended duration of swimming within the target quadrant. These findings indicated that treatment with tilianin improved learning and memory capability in animals with VaD. In parallel with the amelioration of neuronal degeneration by tilianin in the VaD model, *in vitro* tilianin treatment demonstrated protective efficacy in OGD-injured hNCs by increasing survival rate and decreasing neuronal apoptosis as indicated by TUNEL staining. Therefore, it could be inferred that tilianin effectively improved cognition in VaD, possibly through beneficial effects on neurons.

In recent years, excitatory amino acid (EAA) toxicity and subsequent calcium overload have attracted attention in cerebral ischemia and VaD injury [33]. Acute ischemic injury caused increased permeability of the neuronal cell membrane to calcium ions, resulting in intracellular Ca^2+^ overload, including severe neuronal apoptosis and destruction [34]. Calcium overload results in cell damage. On the one hand, it directly causes injury to mitochondrial calcium deposition in neuronal cells, leading to oxidative phosphorylation imbalance and disorders of energy production. On the other hand, increased Ca^2+^ content also activates Ca^2+^-dependent enzymes and phosphorylases, triggering a series of abnormal signaling pathways [35].

CaMKII is an enzyme crucial in the Ca^2+^/CaM signaling pathway, initially activated by EAA and calcium overload during acute cerebral ischemia [36]. Consistent with this, previous results demonstrated that *in vitro* transient OGD injury upregulated CaMKII expression in SH-SY5Y cells [15]. However, different presentations of the active isoforms of CaMKII were identified in the 2VO rat model, with p-CaMKII downregulated while ox-CaMKII upregulated. One possible explanation of the VaD-mediated decrease in p-CaMKII levels was modulation of positive feedback by CREB upstream regulators such as ERK1/2. As the VaD model represents both chronic and long-term ischemia, neuronal cells experience long-term ischemic injury and dysfunction of neurotransmission [35]. Thus, long-term memory-associated signaling pathways such as the CaMKII/ERK/CREB pathway become inactivated, leading to cognitive impairment.

Once CaMKII is phosphorylated by intracellular calcium ions, with autophosphorylation maintained by catalytic activity, it becomes a “molecular memory switch” that continuously activates downstream signaling molecules that regulates nuclear gene expression and maintains cognitive function [5]. ERK1/2 belongs to MAPK family members, functioning as a highly conserved kinase linking phosphorylated CaMKII to its downstream effector [37, 38]. CREB is a phosphorylation substrate of phosphorylated ERK1/2, responsible for gene transcription of dendritic development and synapse formation [39, 40]. In the present study, treatment with tilianin was exhibited to alleviate learning and memory impairment caused by VaD, the therapeutic effect of which may be ascribed to greater memory-related signaling in the hippocampus. Considering the weak activation of CaMKII kinase activity by tilianin [15, 41], we speculated that tilianin might enhance the levels of p-CaMKII in the hippocampus and then display a functional restoration of phosphorylated CaMKII/ERK/CREB signaling transduction rather than altering total CaMKII protein expression. This was verified by the increased levels of p-CaMKII and the downstream upregulation of phosphorylation of ERK1/2/CREB in long-term tilianin treatment in rats with 2VO. Therefore, the preservation of phosphorylated CaMKII/ERK/CREB signaling may be reasonably interpreted as a cognitive improvement by tilianin when treating VaD.

In parallel with the pathology of learning acquisition and hippocampus-dependent memory in VaD, oxidative stress imbalance caused by decreased levels of the antioxidant enzymes SOD and GSH-Px and increased levels of MDA, 4-HNE, and 3-NT occurred due to the process of chronic cerebral ischemia. It is known that intracellular calcium overload causes mitochondrial dysfunction, and mitochondrial calcium ion uptake promotes ROS production [42, 43]. GSH and SOD are among the most important physiological antioxidants against free radicals, preventing subsequent lipid peroxidation and protein oxidation [44]. When ROS and membrane lipids undergo lipid peroxidation reaction, a large quantity of lipid peroxidation products is produced, including MDA and 4-HNE, which are the markers that indicate the degree of lipid peroxidation [45]. ROS reacts with protein amino acid residues to produce protein oxidation products, among which 3-NT is a commonly used detection indicator [46]. Furthermore, studies have observed increased MDA and 4-HNE levels and decreased SOD levels in VaD patients [47]. This study found similar changes of these oxidative markers in the cortical tissue of rats with 2VO and the human neurons suffering from OGD. Combined with the previous finding that tilianin eliminates excessive ROS of the mitochondrion in OGD-injured SH-SY5Y cells [15], the antioxidant effect of tilianin against VaD injury may be attributed to increased enzyme activity and decreased oxidative stress products.

Significantly, excessive ROS created ox-CaMKII, which in turn triggered a series of downstream signaling. Previous studies of myocardial ischemia indicate that ox-CaMKII enhances NF-*κ*B-mediated inflammatory signaling in cardiomyocytes and is closely associated with the development of cardiovascular and pulmonary diseases and cancer [7, 48]. We demonstrated that ox-CaMKII was associated with an inflammatory response mediated by downstream p38 MAPK/JNK-NF-*κ*B in both ischemic cardiomyocytes and neurons [15, 41]. In the present study, tilianin treatment had the effect of inhibiting the expression of ox-CaMKII, in addition to suppressing the levels of p-p38, p-JNK, p-p65, and inflammatory factors TNF-*α*, IL-6, and IL-1*β* both *in vivo* and *in vitro*. Accordingly, the beneficial action of tilianin in ischemic hNCs was blocked when CaMKII*α* siRNA was present, preventing the preservation of neurons and the suppression of p38 MAPK/JNK-NF-*κ*B signaling as experienced with tilianin.

The cellular mechanisms that occur in neuronal injury in VaD are complex, involving oxidative stress, inflammation, and cellular apoptosis, playing a crucial role in neurodegeneration. The hippocampus is principally associated with long-term memory, so neuronal damage is the cause of memory loss and cognitive impairment in VaD. Studies have demonstrated that apoptotic neurons account for a greater proportion of those that die in VaD [49]. Our results also indicated that the ratio of the apoptotic factors Bcl-2/Bax in the intrinsic apoptosis pathway in the hippocampus of rats with 2VO was severely reduced, while the caspase-3 activity increased. However, tilianin treatment guarded against these changes but became uncontrolled by tilianin when cultures were supplemented with CaMKII*α* siRNA. Together, these results suggest that tilianin suppressed neuronal intrinsic apoptosis pathways in VaD via inhibition of CaMKII*α*.

Despite the encouraging results in the present study, there are several limitations. Firstly, diverse models of VaD should be established to clarify the pharmacological effects of tilianin. Secondly, more specific mechanism confirmation on CaMKII signaling pathways should be investigated *in vivo* to elucidate the action of tilianin against VaD.

## 5. Conclusions

**Figure 13:**
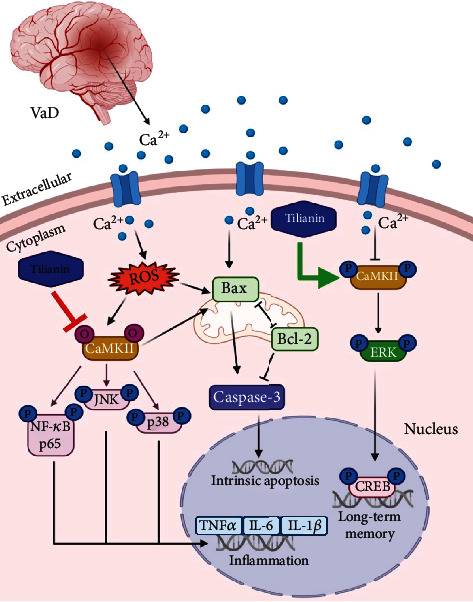
Potential neuroprotective mechanism of tilianin against VaD via the p-CaMKII and ox-CaMKII signaling pathways as established in the present study. Tilianin promoted long-term memory-related signaling pathways and inhibited apoptosis, inflammatory reaction, and oxidative stress levels via CaMKII*α*. Bax: B cell lymphoma-2 associated X protein; Bcl-2: B cell lymphoma-2; CaMKII: Ca^2+^/calmodulin-dependent protein kinase II; caspase: cysteine-dependent aspartate-specific proteases; CREB: cAMP-response element-binding protein; ERK: extracellular regulated protein kinases; IL-1*β*: interleukin-1 beta; IL-6: interleukin-6; JNK: c-Jun N-terminal kinase; NF-*κ*B: nuclear factor kappa-B; ROS: reactive oxygen species; TNF-*α*: tumor necrosis factor-alpha; VaD: vascular dementia.

In conclusion, the present study suggests that tilianin has ameliorative effects on cognitive deficits and neurodegeneration in VaD. Furthermore, the potential therapeutic effects of tilianin against VaD may be associated with the dual CaMKII targeting action of increased p-CaMKII/ERK/CREB and decreased ox-CaMKII/MAPK/NF-*κ*B (Figure 13). Therefore, tilianin may represent a promising novel protective strategy for the treatment of VaD.

## Figures and Tables

**Table 1:** Primary antibodies used in Western blot analysis.

Primary antibody	Dilution	Source
Anti-oxidized-CaMKII (Met281/282) rabbit pAb	1 : 1000	GeneTex
Anti-phospho-CaMKII*α* (Thr286) rabbit pAb	1 : 1000	Abcam
Anti-CaMKII rabbit mAb	1 : 1000	Abcam
Anti-CaMKII*α* rabbit mAb	1 : 10000	Abcam
Anti-Bcl-2 rabbit pAb	1 : 500	Proteintech
Anti-Bax rabbit mAb	1 : 500	CST
Anti-phospho-SAPK/JNK (Thr183/Tyr185) mouse mAb	1 : 2000	CST
Anti-JNK rabbit pAb	1 : 1000	Abcam
Anti-phospho-p38 MAPK (Thr180/Tyr182) rabbit mAb	1 : 1000	CST
Anti-p38 MAPK rabbit mAb	1 : 1000	Abcam
Anti-phospho-p44/42 MAPK (Thr202/Tyr204) rabbit mAb	1 : 2000	CST
Anti-p44/42 MAPK rabbit mAb	1 : 1000	Abcam
Anti-phospho-CREB (Ser133) rabbit mAb	1 : 1000	Abcam
Anti-CREB rabbit mAb	1 : 1000	CST
Anti-phospho-NF-*κ*B p65 (Ser536) rabbit mAb	1 : 1000	CST
Anti-NF-*κ*B p65 rabbit pAb	1 : 1000	Abcam
Anti-phospho-IKK*α*/*β*(Ser176/180) rabbit mAb	1 : 1000	CST
Anti-IKK*α*/*β* rabbit mAb	1 : 1000	CST
Anti-I*κ*B*α* rabbit mAb	1 : 1000	Abcam
Anti-GAPDH rabbit pAb	1 : 5000	Proteintech

Note: GeneTex, Irvine, CA, USA; Abcam, Cambridge, MA, USA; Proteintech, Rosemont, IL, USA; CST, Cell Signaling Technology, Danvers, MA, USA.

**Table 2:** Sequences of siRNAs used for cell transfection.

siRNA name	siRNA sequence
Negative control (NC)	Sense: 5′-UUCUCCGAACGUGUCACGUTT-3′
	Antisense: 5′-ACGUGACACGUUCGGAGAATT-3′

CaMKII*α*	Sense: 5′-GGACCUGAAGCCUGAGAAUCUTT-3′
	Antisense: 5′-AGAUUCUCAGGCUUCAGGUCCTT-3′

**Table 3:** Primary antibodies used in cell immunofluorescence assay.

Primary antibody	Dilution	Source
Anti-oxidized-CaMKII (Met281/282) rabbit pAb	1 : 50	GeneTex
Anti-Bcl-2 rabbit pAb	1 : 200	Proteintech
Anti-Bax rabbit mAb	1 : 200	CST
Anti-phospho-NF-*κ*B p65 (Ser536) rabbit mAb	1 : 100	CST

Note: GeneTex, Irvine, CA, USA; Proteintech, Rosemont, IL, USA; CST, Cell Signaling Technology, Danvers, MA, USA.

## Data Availability

The data used to support the findings of this study are available from the corresponding authors upon request.

## Supplementary Materials

Supplementary Materials Figure S1: immunofluorescence staining of characteristic proteins in human neural stem cells (hNSCs) and human nerve cells (hNCs). Figure S2: expression of CaMKII*α* after transfection with siRNA. Figure S3: whole uncropped images of the original Western blot bands with five repetitions for Figure 6. Figure S4: whole uncropped images of the original Western blot bands with five repetitions for Figures 8(a)–8(c). Figure S5: whole uncropped images of the original Western blot bands for Supplementary Figure 2. Table S1: qPCR primer sequences.
