# Supplementary material for: Tilianin Ameliorates Cognitive Dysfunction and Neuronal Damage in Rats with Vascular Dementia via p-CaMKII/ERK/CREB and ox-CaMKII-Dependent MAPK/NF-κB Pathways
Source: Oxid Med Cell Longev. 2021 Sep 4;2021:6673967. doi: 10.1155/2021/6673967 (PMC8437593; doi:10.1155/2021/6673967)
Supplement: Supplementary Materials — Figure S1: immunofluorescence staining of characteristic proteins in human neural stem cells (hNSCs) and human nerve cells (hNCs). Figure S2: expression of CaMKIIα after transfection with siRNA. Figure S3: whole uncropped images of the original Western blot bands with five repetitions for Figure 6. Figure S4: whole uncropped images of the original Western blot bands with five repetitions for Figures 8(a)–8(c). Figure S5: whole uncropped images of the original Western blot bands for Supplementary Figure 2. Table S1: qPCR primer sequences. [file 6673967.f1.docx]

**Supplementary Materials**


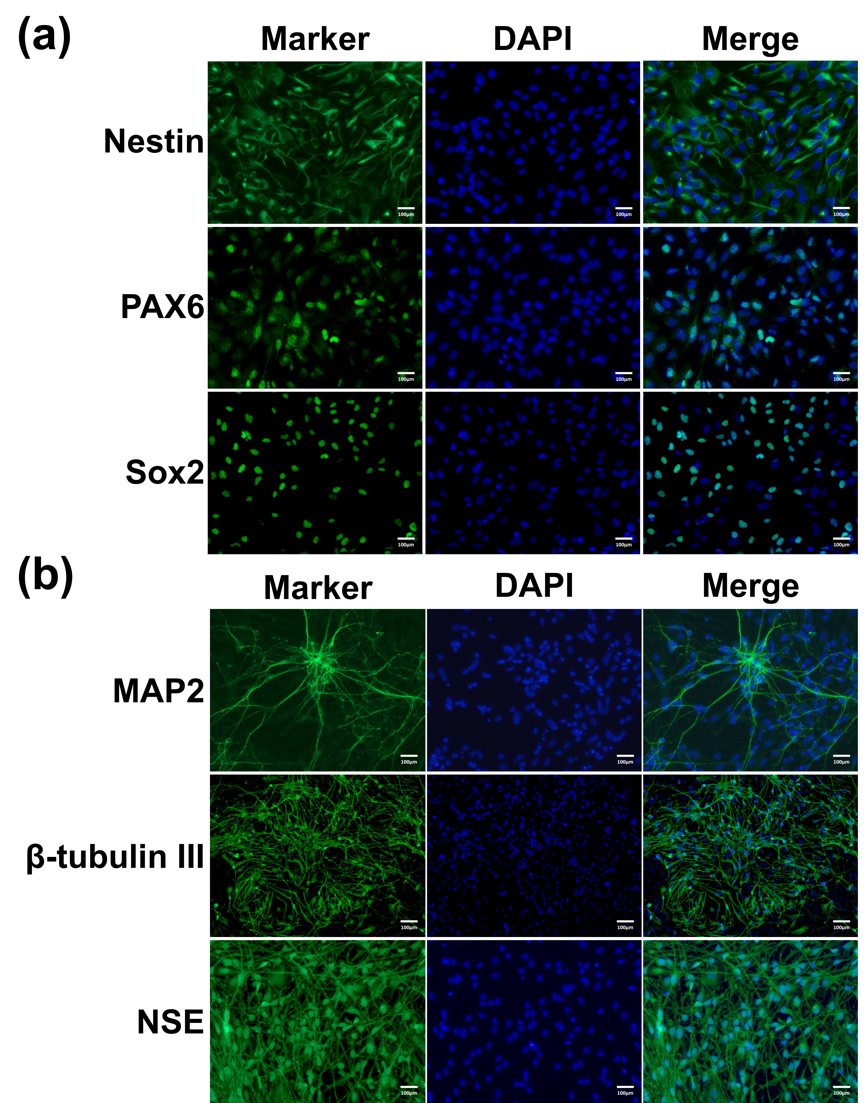


**Supplementary Figure 1:** **Immunofluorescence staining of characteristic proteins in human neural stem cells (hNSCs) and human nerve cells (hNCs).** (a) Representative images of Nestin, PAX6, and Sox2 staining of hiPSC-derived NSCs. (b) Representative images of MAP2, β-tubulin III, and NSE staining of hNCs with 21 days differentiation. Bar: 100 μm.


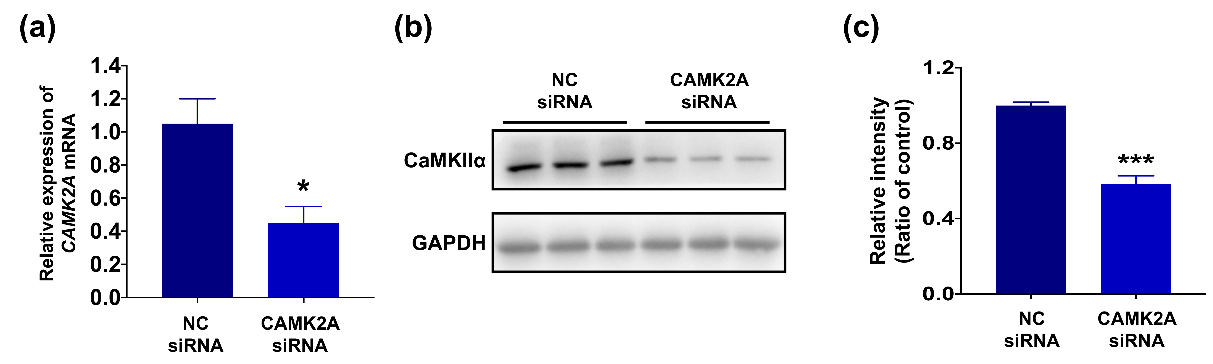


**Supplementary Figure 2: Expression of CaMKIIα after transfection with siRNA.** (a) Quantiﬁcation of *CAMK2A* mRNA levels after siRNA transfection. (b) Representative Western blot images of CaMKIIα after transfection with siRNA. (c) Quantitative analyses for Western blot bands of CaMKIIα. Results are expressed as mean ± SD, *n* = 3. ^*^*P* < 0.05, ^***^*P* < 0.001 *vs.* NC siRNA.

**Supplementary Table 1:** qPCR primer sequences.

| **Primer Name** | **Primer Sequence** |
| --- | --- |
| **CAMK2A-F** | 5’- CCAAAGTGCGGAAACAGGAA -3’ |
| **CAMK2A-R** | 5’- TCGCACATCTTCGTGTAGGA -3’ |
| **ACTB-F** | 5’- TGGACTTCGAGCAAGAGATG -3’ |
| **ACTB-R** | 5’- GAAGGAAGGCTGGAAGAGTG -3’ |


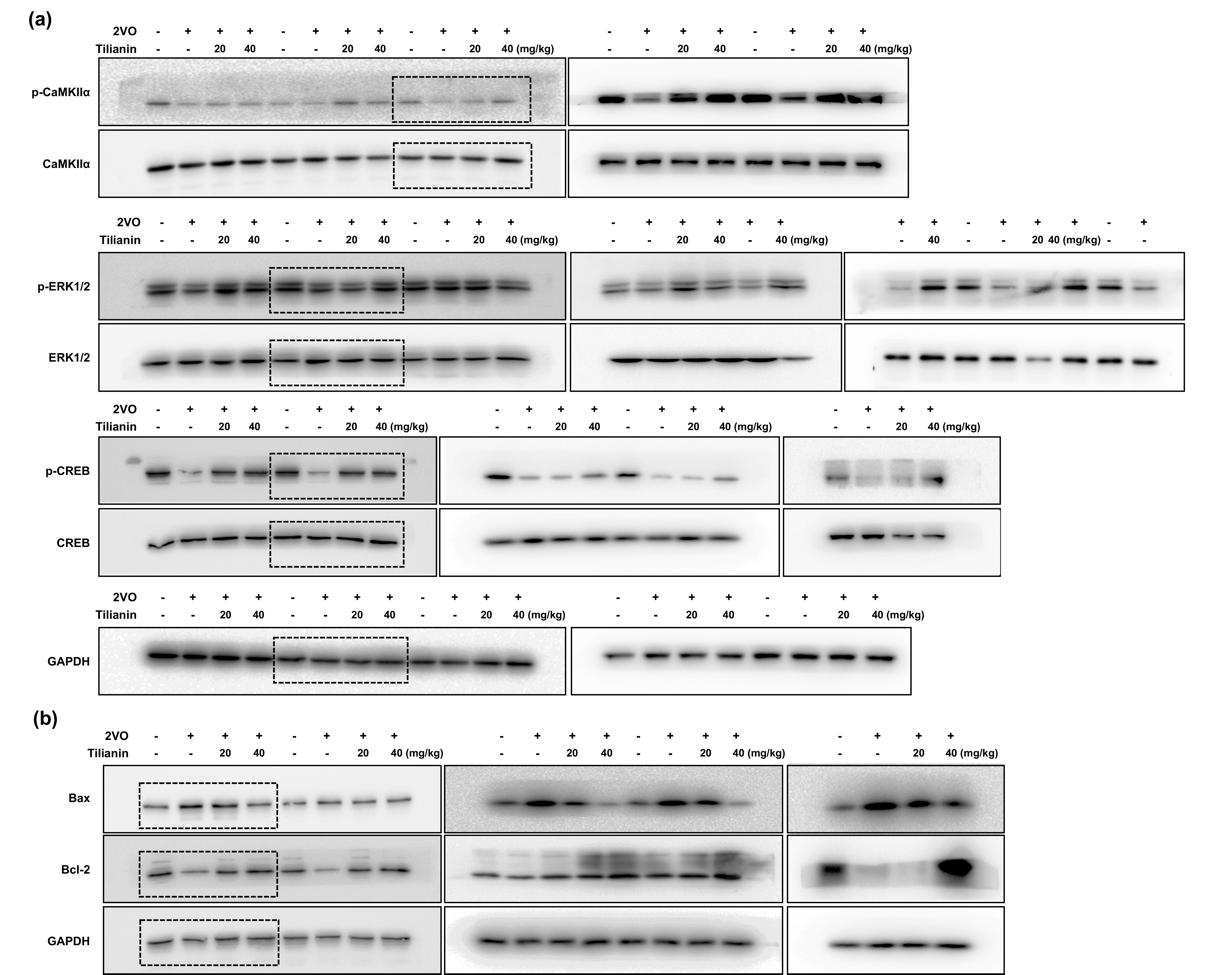


**Supplementary Figure 3:** **Whole uncropped images of the original Western blot bands with five repetitions for Figure 6.** (a) Whole uncropped images of the original Western blot bands with five repetitions used in Figure 6(a) and Figure 6(b). (b) Whole uncropped images of the original Western blot bands with five repetitions used in Figure 6(c) and Figure 6(d). The representative bands used are marked with boxes.


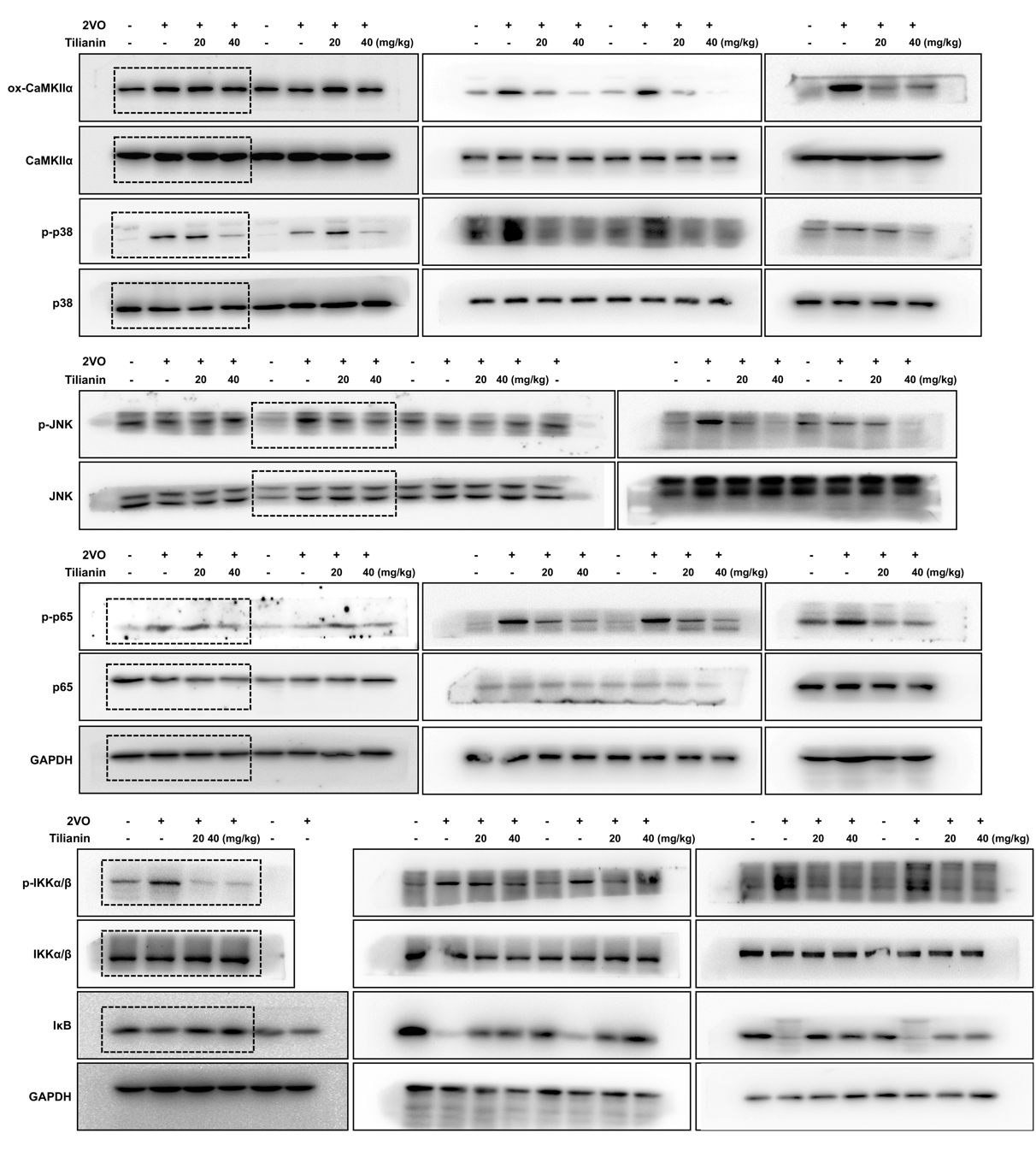


**Supplementary Figure 4:** **Whole uncropped images of the original Western blot bands with five repetitions for Figure 8(a)**-**(c).** The representative bands used are marked with boxes.


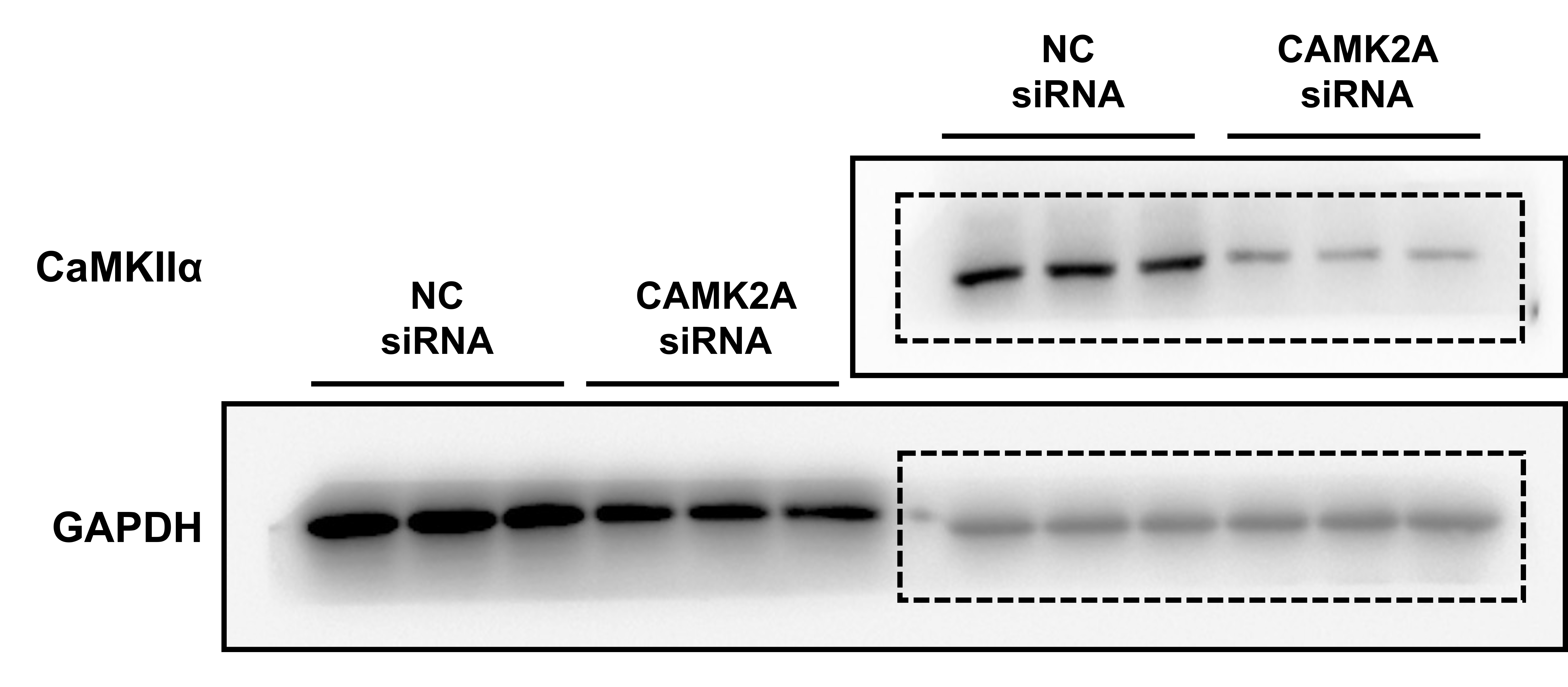


**Supplementary Figure 5:** **Whole uncropped images of the original Western blot bands for Supplementary Figure 2.** The representative bands used are marked with boxes.
